# Preoperative diagnosis of bicipitoradial bursitis: a case report

**DOI:** 10.11604/pamj.2014.17.41.3098

**Published:** 2014-01-21

**Authors:** Asim Aldhilan

**Affiliations:** 1 Medical Imaging Department King Abdul Aziz Medical City for National Guard, Riyadh 11426, Saudia Arabia

**Keywords:** Bicipitoradial bursitis, bursa, elbow pain, MRI

## Abstract

Inflammation of the bicipitoradial bursa is a rare condition and only few reports can be found in literature. Several causes for a cubital bursitis have been suggested in the past. The need to include a malignant lesion in the differential diagnosis has only been mentioned in one of these reports. May main objective in reporting this case is to make this pathological entity better known.

## Introduction

Bicipitoradial bursitis refers to inflammation of the bicipitoradial bursa which is located between the distal biceps tendon and the tuberosity of the radius. The bursa partially or completely wraps around the biceps tendon. It ensures frictionless motion between the biceps tendon and the proximal radius during pronation and supination of the forearm. The bicipitoradial bursa surrounds the biceps tendon in supination. In pronation, the radial tuberosity rotates posteriorly, which compresses the bicipitoradial bursa between the biceps tendon and the radial cortex which consequently increases the pressure within the bursa.

## Patient and observation

**Figure 1:**
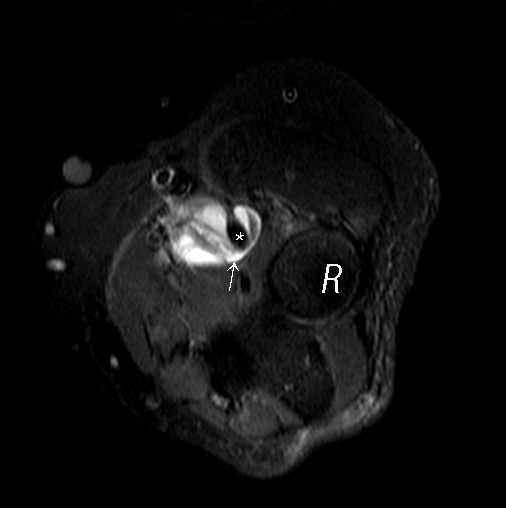
Axial STIR of the elbow shows lobulated mass (arrow) noted between biceps tendon (star) and radial head (R)

**Figure 2:**
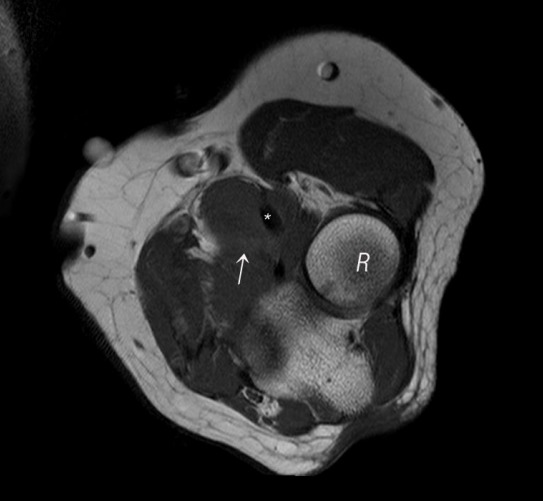
Axial T1 of the elbow shows lobulated mass (arrow) noted between biceps tendon (star) and radial head (R)

**Figure 3:**
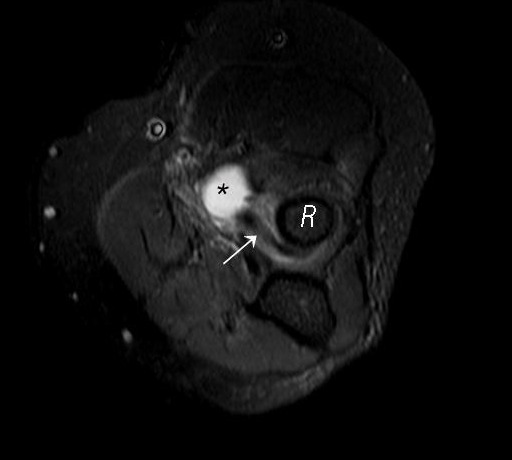
Axial STIR of the elbow shows lower end of biceps tendon (arrow) inserted at the radial head (R). Note the inflamed bursa (star) anterior to the radial head

**Figure 4:**
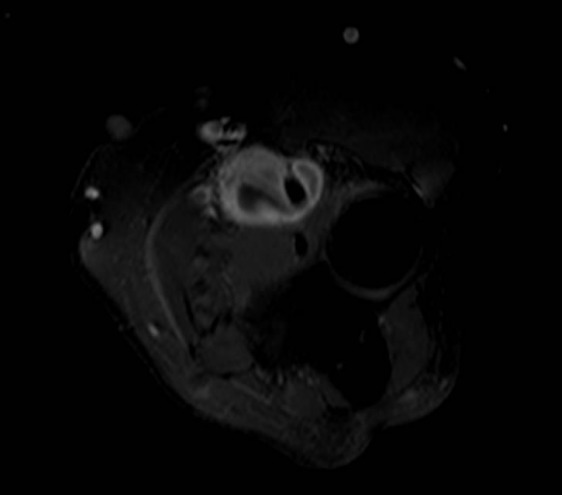
Axial T1 post contrast administration shows lobulated mass with thick peripheral enhancement between biceps tendon and radial head

A 52-year-old, otherwise healthy women was referred to orthopedic clinic with a 3-months history of anterior right elbow pain. She complained of a painful swelling in the right antecubital fossa. There is no history of trauma nor any medical illnesses. On examination there is hard tender soft tissue mass in the right antecubital fossa (Figure 1, Figure 2, Figure 3, Figure 4).

MRI was done and it demonstrate lobulated mass noted adjacent to biceps tendon insertion at radial head measuring 4 x 2 x 2 cm (CC x transverse x AP diameter respectively) post contrast images shows thick peripheral enhancement and internal enhancing septa. Brachial artery and superficial radial nerve located anteromedial and superficial radial artery located lateral to the lesion. Elbow is intact with no joint effusion and visualized bone structures are unremarkable.

Surgical exploration, removal of the lesion and histological investigation provided definite diagnosis and treatment. The bursa was wrapped around the distal biceps tendon with clear margins between both structures. Histological investigations confirmed the diagnosis of an inflamed bicipitoradial bursa.

## Discussion

Bursae are frequently interposed between tendons and bone. They allow the tendons to glide smoothly over the bony surface. The elbow's cubital fossa has two bursae: the bicipitoradial bursa and the interosseous bursa. The bicipitoradial bursa is located between the distal biceps tendon and the tuberosity of the radius. It partially or completely wraps around the biceps tendon. It ensures frictionless motion between the biceps tendon and the proximal radius during pronation and supination of the forearm [2].

Inflammation of the bicipitoradial bursa is a rather rare condition, of which only few reports can be found in orthopaedic literature [1, 2, 4, 5]. Several causes for bursitis at this location are suggested, such as repetitive mechanical trauma or overuse [4, 2, 6], chemical or infectious synovitis [1, 2, 7], rheumatic disease [2, 8], partial tear of biceps tendon [2, 3], synovial cyst at the sacciform recess of the anterior elbow capsule [1] and synovial chondromatosis [5].

Clinically cubital bursitis almost always presents as a painful mass in the proximal forearm, somewhat restricting elbow motion. With pronation, the tuberosity of the radius rotates posteriorly, causing compression of the bursa between the biceps tendon and the radial tuberosity [2, 8]. In severe cases the mass may compress the radial nerve. Compression of the deep radial nerve can result in weakness of the extensor muscles of the forearm. Compression of the superficial ramus of the radial nerve may result in sensory loss at the dorsum of the hand and fingers. The median nerve is unlikely to be compressed because of its medial position relative to the bursa [2, 7, 9].

Accurate diagnosis of bicipitoradial bursitis requires imaging studies. MR is the imaging investigation of choice for studying lesions in the antecubital fossa. It not only demonstrates the relationship between the bursa and adjacent structures, but is also of great value in distinguishing the bursa from other mass lesions in this area, such as ganglion cysts and tumors [2, 7].

Treatment is based on the patient's symptoms with a tendency towards surgical removal in case of continuing pain in spite of conservative measures or in the presence of nerve compression and/or restriction of movement. Conservative treatment consists of anti-inflammatory medication, relative rest or temporary cast immobilisation [2]. Aspiration of the bursa and injection of a corticosteroid can be considered [10].

## Conclusion

Bicipitoradial bursitis is one of the rare causes of anterior elbow pain. The anatomy of the bicipitoradial bursa is best evaluated by MR imaging. MR imaging allows accurate diagnosis of bicipitoradial bursitis and its effects on adjacent structures.
